# Supplementary material for: Using strain to uncover the interplay between two- and three-dimensional charge density waves in high-temperature superconducting YBa2Cu3Oy
Source: Nat Commun. 2024 Apr 16;15:3277. doi: 10.1038/s41467-024-47540-w (PMC11021565; doi:10.1038/s41467-024-47540-w)
Supplement: Supplementary file 1 — Supplementary Information [file 41467_2024_47540_MOESM1_ESM.pdf]

Supplementary Information

Using strain to uncover the interplay between two- and three-dimensional charge density waves in high-temperature superconducting $YBa_2Cu_3O_y$

I. Vinograd,[1,2,*] S. M. Souliou,[1,*] A.-A. Haghighirad,[1] T. Lacmann,[1] Y. Caplan,[3] M. Frachet,[1] M. Merz,[1,4] G. Garbarino,[5] Y. Liu,[6] S. Nakata,[6] K. Ishida,[7,†] H. M. L. Noad,[7] M. Minola,[6] B. Keimer,[6] D. Orgad,[3] C. W. Hicks,[7,8] and M. Le Tacon[1,‡]

[1]*Institute for Quantum Materials and Technologies, Karlsruhe Institute of Technology, Kaiserstr. 12, D-76131 Karlsruhe, Germany*

[2]*4th Physical Institute – Solids and Nanostructures, University of Göttingen, D-37077 Göttingen, Germany*

[3]*Racah Institute of Physics, The Hebrew University, Jerusalem 91904, Israel*

[4]*Karlsruhe Nano Micro Facility (KNMFi), Karlsruhe Institute of Technology, Kaiserstr. 12, D-76131 Karlsruhe, Germany*

[5]*ESRF, The European Synchrotron, 71, avenue des Martyrs, CS 40220 F-38043 Grenoble Cedex 9*

[6]*Max Planck Institute for Solid State Research, Heisenbergstraße 1, D-70569 Stuttgart, Germany*

[7]*Max Planck Institute for Chemical Physics of Solids, Nöthnitzer Str. 40, D-01187 Dresden, Germany*

[8]*School of Physics and Astronomy, University of Birmingham, Birmingham, B15 2TT, UK*

(Dated: March 28, 2024)

Supplementary Note 1. Experimental details

The single crystal samples were grown by a flux technique described in Ref.[1] and the sample information is summarized in Supplementary Table 1. Superconducting T_c and c axis parameters were used to determine the hole doping. The samples were glued into a titanium cross carrier using Stycast FT-2850. A frame was screwed onto the Razorbill CS200T strain cell into which the cross itself was glued. This allows us to perform the delicate step of gluing the sample onto the cross prior to the experiments. The nominal stress application is monitored by a capacitive sensor integrated in the cell. The capacitance is monitored with an AH2550A high-resolution

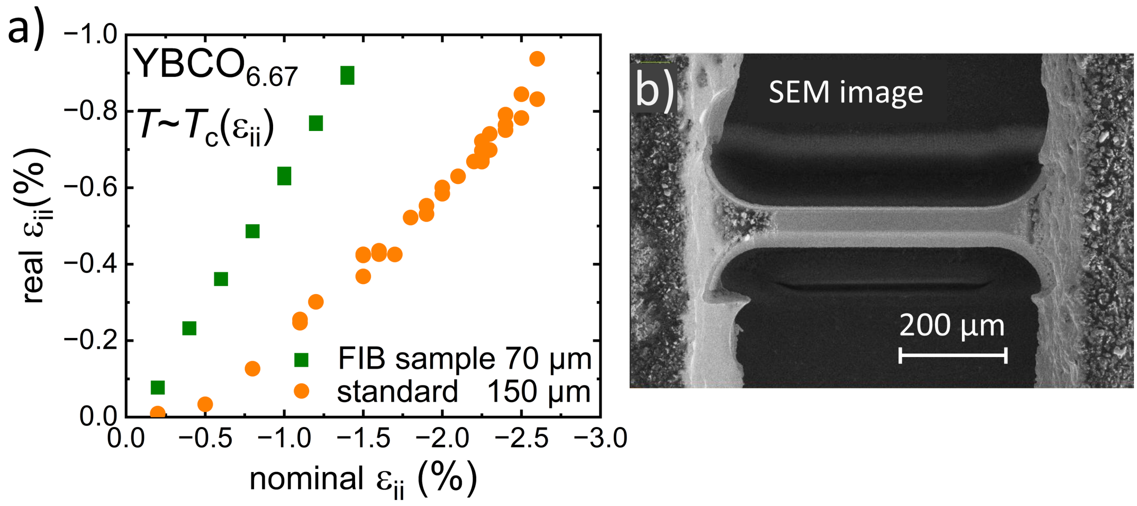

Supplementary Figure 1: (a) Strain transmission is estimated from real versus nominal strain for a standard sample (orange circles, a-compression) and a sample thinned to 70 μm using a Xe-plasma FIB (green squares, b-compression). The FIB sample displays a better strain transmission. Error bars of the real strain from CrysalisPro are smaller than the symbol size. (b) SEM image of the thinned sample after the FIB processing.

capacitance bridge or a Keysight 4980AL LCR meter and converted into a nominal displacement and strain. The effective strain transmission is estimated from the slope in Supplementary Fig. 1. Reducing the sample width using a Xenon plasma focused ion beam (Xe-plasma FIB) improves the strain transmission significantly, but the maximally achieved real strain remains just above -1 %, a value beyond which samples typically break. We confirmed by SQUID measurements that the FIB processing does not modify the sample's bulk T_c.

oxygen content y	doping p	T_c (K)	compression & transmission axis
6.67	0.125	67	a & b axis
6.67	0.125	67	b & a axis
6.55	0.108	61	a & c axis
6.67	0.125	67	b & c axis (cut with FIB)
6.80	0.140	78	a & c axis

Supplementary Table 1: Sample information and sample geometry (compression axis and crystal axis parallel to beam during transmission at zero rotation)

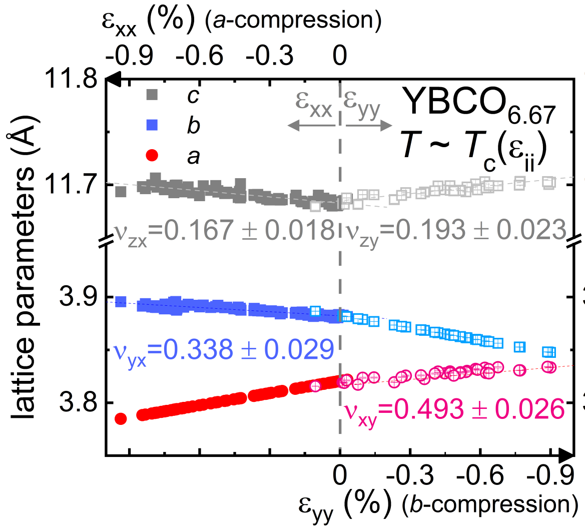

Supplementary Figure 2: Strain evolution of the unit cell parameters for a and b axis compressions of the three investigated $YBCO_{6.67}$ samples. Error bars are determined by CrysalisPro. For linearly expanding transverse directions the Poisson ratios are determined from the linear intercept and the slope.

The high-pressure beamline ID15B at ESRF operates with monochromatized 30 keV photons (λ=0.4101 Å) produced by an undulator[2]. The flux is reduced by increasing the undulator gap which shifts the harmonics energy relative to the monochromator. This allows to prevent beam-heating of the sample in high flux measurements and not to over-saturate the lattice Bragg peaks in low flux measurements. A side-effect of the increased undulator gap is the relative increase in intensity of undesired harmonics which pass the monochromator at $\lambda/2$. This leads to weaker but visible lattice Bragg peaks appearing at half-integer reciprocal lattice values. These $\lambda/2$ peaks are too weak to be visible in the lowest flux data (undulator gap = 15.6-16.6 mm). Higher flux (undulator gap = 13.5 mm) saturates the main lattice Bragg reflections in all presented reciprocal space maps. The sharp $\lambda/2$ peaks can be easily distinguished from CDW peaks from their temperature and strain dependence. The sharpness of the $\lambda/2$ peaks in $(0KL)$ and $(H0L)$ maps are evidence of the high crystal quality, even when the main lattice peaks are over-saturated and appear very broad.

Since we observe a linear expansion of the transverse crystallographic axes in Supplementary Fig. 2, described by slope m and intercept b, we can assume a strain-independent Poisson ratio and calculate the Poisson ratio as $\nu_{ij} = -m/b \times 100\%$. The systematic uncertainty in the determination of $\varepsilon_{ii} = 0$ due to differences in the thermal expansions of the strain cell and the

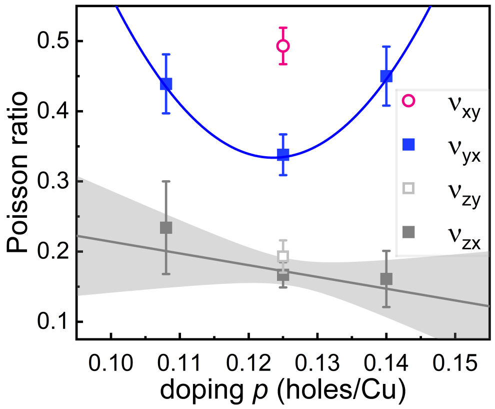

Supplementary Figure 3: Hole doping dependence of the Poisson ratios for the investigated samples with one-σ confidence intervals for the linear fit of ν_{zx} indicated by shaded grey regions. This one σ-confidence interval of the fit indicates that ν_{zx} can be assumed as doping independent up to the experimental precision. The parabola is a guide to the eye for ν_{yx}. Empty symbols correspond to ν_{xy} and ν_{zy} determined from b axis compression. Error bars result from standard deviations of the linear fits in Supplementary Fig. 2 and in Supplementary Fig. 6.

sample after cooling down is estimated to lie within ±0.05%, as witnessed from the intercept of linear fits for a and b axis compression in Supplementary Fig. 2 being close to $\varepsilon_{ii} = 0$. That a and b axis compression lead to $\nu_{yx} \neq \nu_{xy}$, for the $YBCO_{6.67}$ samples in Supplementary Fig. 3 is expected for the orthorhombic lattice. It means that the Young's moduli, E_i are different, following $\frac{\nu_{yx}}{E_y} = \frac{\nu_{xy}}{E_x}$, however, ∼47% difference is remarkable.

Supplementary Note 2. 2D and 3D CDWs

Our study mainly focuses on the 2D vs. 3D CDW competition in $YBCO_{6.67}$, as at this doping the 2D CDW is best developed and was consequently studied extensively in previous x-ray diffraction studies[3–5]. In addition, our measurements of the $YBCO_{6.55}$ sample, are consistent with the scenario of direct competition between 2D and 3D CDWs, as shown in Supplementary Figs. 4b,c. Similarly to $YBCO_{6.67}$, the 2D and 3D CDW intensities increase for a axis compression, but the 2D CDW intensity does not show a cusp at the 3D CDW onset. Only after further compression where the 3D intensity starts to grow more rapidly, as can be seen by the increased linear slope of the growing intensity, the 2D CDW intensity drops. This behavior is illustrated for two datasets: i) The temperatures for which the 3D CDW intensity is maximal, thus likely being close to $T_c(\varepsilon_{xx})$, which decreases with a axis compression. ii) Fixed temperature at

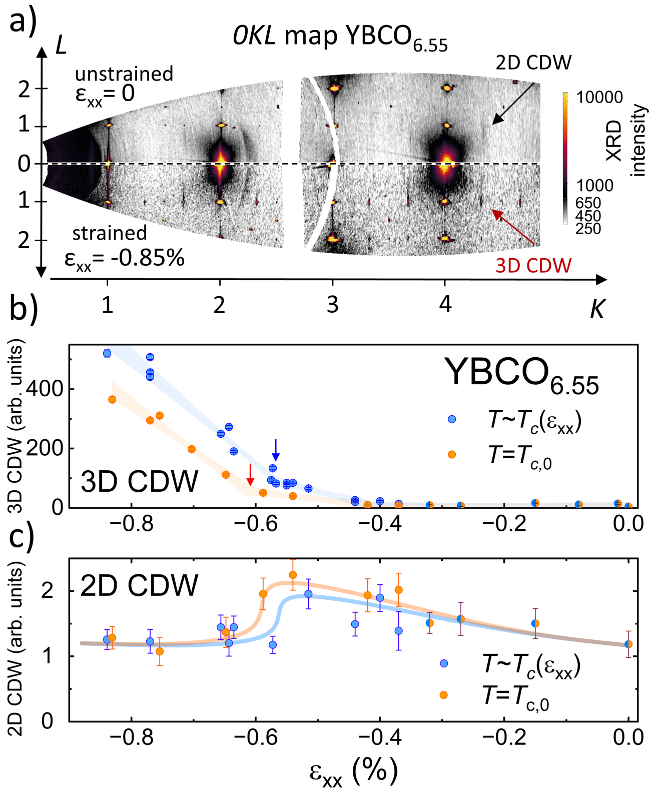

Supplementary Figure 4: (a) $YBCO_{6.55}$ $(0KL)$ reciprocal space maps at zero (top) and highest a axis compression (bottom) measured at $T_c(\varepsilon_{xx}=0)$=61 K, show the appearance of sharp 3D CDW peaks at integer L at high compression. Faint 2D CDW intensity in between is present at all strains close to half-integer L. (b) Strain dependence of the integrated 3D b-CDW peak at $(K,L)=(4+k_{\mathrm{CDW}},-1)$ from L-cuts is maximal at $T\sim T_c(\varepsilon_{xx})$ (blue circles). For comparison, the 3D CDW intensity is shown at constant temperature of $T_c(\varepsilon_{xx}=0)$=61 K. Lines are piece-wise linear fits (widths correspond to one-σ confidence intervals), showing the onset of 3D intensity at strains as low as ε_{xx}=-0.4% and arrows mark an inflection point, beyond which 3D order grows more rapidly. (c) Strain dependence of the integrated 2D b-CDW peak at $(K,L)=(4-k_{\mathrm{CDW}},0.5)$ from K-cuts shows decreasing 2D CDW intensity beyond the strain value where 3D CDW order grows rapidly. Lines are guides to the eye. Error bars correspond to standard deviations of Lorentzian fits.

$T_c(\varepsilon_{xx}=0)$=61 K of the unstrained sample. It is interesting to note that the 3D CDW is already well developed in the intermediate strain range $-0.55\% \leq \varepsilon_{xx} \leq -0.40\%$ and the maximal peak intensity of the 3D CDW exceeds that of the broad 2D CDW multiple times. Whereas the peak intensity of the 2D CDW, I^{2D}_{max} is of the order of 100 counts in our measurements for $YBCO_{6.55}$ and $YBCO_{6.67}$, the peak intensity of the 3D CDW, I^{3D}_{max}, is $\sim$ 500 counts in the intermediate strain range. The resulting relative 3D peak intensity $I^{3D}_{max}/I^{2D}_{max} \sim 5$ is thus comparable to

the highest relative peak intensities reached in high fields up to 26 T[5,6]. Based on high field NMR measurements up to 45 T, the 3D CDW is already nearly fully developed around ~ 25 T[7]. This implies that high magnetic fields alone are not able to reach a 3D CDW state of equal magnitude as by applying large a axis compression. This is likely the reason why high field experiments did not observe this fierce competition of 2D and 3D CDWs, although a plateau in the field dependence of 2D b-CDWs is suggestive of a related but weaker effect[5].

How can the faint 2D CDW's impact on the sharp 3D CDW be explained? Whereas the peak intensities of the 2D and 3D CDWs vary dramatically, the total integrated intensities of the two CDWs are rather comparable, within the range of investigated a axis compression. To estimate the total integrated intensities the correlation lengths $\xi_i = 1/\sigma_i$ (σ_i is the standard deviation of a Gaussian in the direction i) need to be known[8]. Assuming a purely Gaussian peak shape in all three directions of the reciprocal space, the total integrated intensity, I_{tot}, is fully determined by the volume fraction occupied by CDWs, v_{frac}, the maximal peak intensity I_{max} and the inverse correlation lengths ξ_H^{-1}, ξ_K^{-1} and ξ_L^{-1}, as $I_{tot} \propto v_{frac}^{-1} \cdot I_{max} \cdot \xi_H^{-1} \cdot \xi_K^{-1} \cdot \xi_L^{-1}$. These values are listed for the $YBCO_{6.67}$ and $YBCO_{6.55}$ samples in Supplementary Table 2 and can be compared to the correlation lengths of the O-VIII and O-II chain order, as defects of the chains are thought to pin 2D CDWs[9]. While these correlation lengths are the relevant quantities to estimate I_{tot} in our experiment, the values discussed here should be considered as lower bounds of the actual correlation length of the 3D CDW order. For both samples, these values are indeed limited by the instrumental resolution (note that it differs for the two samples that were investigated in different scattering geometries).

From experimental data alone, it is difficult to determine whether the CDW's periodic lattice displacement or the volume fraction is responsible for the increasing CDW peak intensities. However, in the case of 3D CDWs, the correlation lengths are so large that the volume fraction is likely to be close to 1, meaning that I_{tot} mainly grows due to an increasing periodic lattice displacement (squared)[10]. To effectively compete with the 3D CDW, the volume fraction of the 2D b-CDW must be large, too. In the theoretical model, its volume fraction is mainly determined by disordered regions due to pinning and is consequently weakly dependent on strain. The 2D CDW peak widths are not changing much with increasing strain, despite the growth in intensity. Hence it is not important whether H-, K- or L-cuts are used to plot 2D or 3D integrated intensities and one can assume that the 2D CDW's intensity is a measure of the periodic lattice displacement, with the correlation lengths ξ_i being approximately limited by

the correlation length of the chain order. Given the large widths of the 2D CDW peaks, large strains are needed for the 3D CDW's periodic lattice displacement to surpass its magnitude for the 2D CDW as measured by I_{tot}[8]. The apparent competition bears evidence that, in some sense, the faint 2D CDW is on a par with the well-developed 3D CDW order. Consequently, this substantiates the notion that 2D CDWs are also strong enough to compete with and can impact the strength of superconductivity itself for dopings close to $p \sim 0.12$, where T_c and especially H_{c2} become anomalously low[11,12]. Thus, the competition between charge density waves (CDW) and superconductivity is not one-sided and dominated by superconductivity.

For the $YBCO_{6.55}$ sample we noted anomalous line shapes of the 3D CDW peaks in L-cuts for which the resolution is worse than for in-plane directions. This could be an artifact of the data processing of CrysalisPro, however we cannot exclude occasional saturation of the detector given the sharpness of the 3D peaks. Still, no anomalies are seen in the line shape shown in Fig. 3 b) of the main manuscript. For unstrained or weakly compressed samples we systematically observe very large secondary extinction factors ~ 1 in refinements with Jana2020. Despite the low flux used, for certain reflections a few pixels of the detector appear to be saturated and

peak	ξ_H (Å)	ξ_K (Å)	ξ_L (Å)	I_{max}	I_{tot}
b-$CDW^{3D}_{6.67}$	143	97	88	10000	$5.8\,10^{-3}$
b-$CDW^{2D}_{6.67}$	29	73	8	110	$4.2\,10^{-3}$
a-$CDW^{2D}_{6.67}$	57	58	8	150	$3.6\,10^{-3}$
$chains_{6.67}$	22	73	8		
b-$CDW^{3D}_{6.55}$	275	274	84	10000	$1.1\,10^{-3}$
b-$CDW^{2D}_{6.55}$	40	75	8	116	$3.1\,10^{-3}$
$chains_{6.55}$	80	182	47		

Supplementary Table 2: Correlation lengths, ξ_i of CDW peaks and chains of the $YBCO_{6.67}$ and $YBCO_{6.55}$ samples, as well as highest achieved (a- or b-compression) I_{max} and corresponding I_{tot}, assuming a CDW volume fraction $v_{frac} = 1$. Due to different experimental geometry (see Supplementary Table 1), the largest accessible CDW peaks are ~3 times weaker for the $YBCO_{6.55}$ sample, as estimated from $YBCO_{6.67}$ at zero strain and transmission through the b axis at zero rotation (large ($0KL$) map).

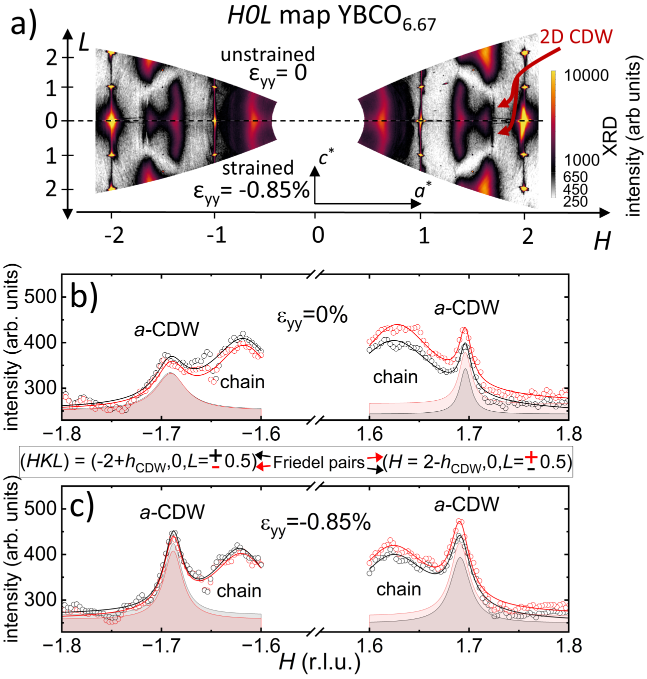

Supplementary Figure 5: $YBCO_{6.67}$ $(H0L)$ reciprocal space maps at zero (top) and highest b axis compression (bottom) measured at $T_c(\varepsilon_{xx}=0)$=67 K, show faint 2D a-CDW intensity at half-integer L close to chain peaks that dominate the $H0L$ map. b) H-cuts across the 2D a-CDW of the unstrained map with anomalous widths for peaks at $H=-2+h_{\mathrm{CDW}}$ and their Friedel pairs at $2-h_{\mathrm{CDW}}$. In each case cuts for $L=\pm0.5$ are shown and Friedel pairs have the same color (red or black). c) Corresponding H cuts through 2D a-CDW peaks at high b axis compression, with similar peaks for both Friedel pairs.

this effect can result in an artificially increased extinction coefficient. For higher compression the lattice peaks broaden slightly and the extinction drops to smaller values. At high flux, the 2D CDW intensity is too weak to saturate the detector, yet measurements of the 2D a-CDW for the $YBCO_{6.67}$ sample are complicated by effects that could be related to extinction. The 2D a-CDW for the $YBCO_{6.55}$ sample was too weak to be studied reliably. In Supplementary Fig. 5b we observe that the peak widths and intensities of the unstrained 2D a-CDW are anomalous. This can be seen from the asymmetric properties of Friedel pairs (peaks related by inversion symmetry). Comparing line cuts in panels b) and c) of Supplementary Fig. 5 one can see that mirror symmetries are re-established and anomalous behavior of Friedel pairs is nearly suppressed at high strain, which potentially modifies the mosaicity of the sample. As the asymmetry is much reduced after compressing the sample, it is not due to an absorption effect but could be related to extinction. Without providing any derivation, we observe a purely

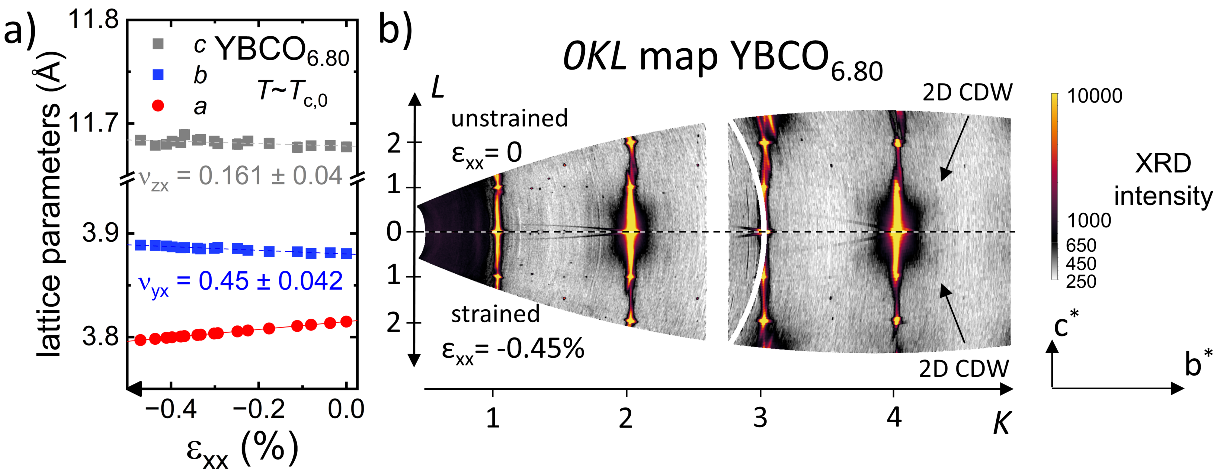

Supplementary Figure 6: (a) Strain evolution of the unit cell parameters for a axis compression of the $YBCO_{6.80}$ sample. (b) $YBCO_{6.80}$ $(0KL)$ reciprocal space maps at zero (top) and highest a axis compression (bottom) measured at 77 K, just below $T_c(\varepsilon_{xx}=0)$=78 K, show no visible effect from the compression at the wave vector of the 2D CDW. The lattice peaks are elongated due to worse sample quality, but this effect is strongly exaggerated due to the chosen upper cut-off when plotting the XRD intensity.

heuristic scaling between the square root of the extinction with the difference of the widths of 2D a-CDW Friedel pairs, $\sqrt{\text{extinction}} \propto \Delta w$ where Δw is the difference of the widths of the a-CDW peaks, shown in Supplementary Fig. 5b,c. For small strains the small and large widths correspond to the correlation lengths $\xi_{a,>} = 110 \pm 18$Å and $\xi_{a,<} = 47 \pm 7$Å. $\xi_{a,<}$ compares better to the longitudinal a-CDW correlation length of 41 ± 1.7Å in ref.[13] and follows the expectations that ξ_a should increase for b axis compression. It is unclear why a-CDW peaks should have different widths, so the associated correlation lengths should be treated with caution.

Similar anomalous effects are also seen for the 2D a-CDW of the measured $YBCO_{6.80}$ sample. However, we focus on the 2D b-CDW, which is expected to increase with a axis compression. In the $(0KL)$ map of Supplementary Fig. 6b we observe no visible effect of the strain on the b-CDW. Consequently, the strain sensitivity must be reduced at this doping, although the negative pressure derivative, $\frac{dT_c}{dp_a} < 0$, is suggestive of the contrary[14]. Possibly, the higher disorder at this doping is problematic and the 3D CDW is reached only if the superconducting T_c is reduced to values comparable to the onset temperature of the negative Hall number ($T_0(p = 0.14) \sim 50$ K) in high field experiments[15].

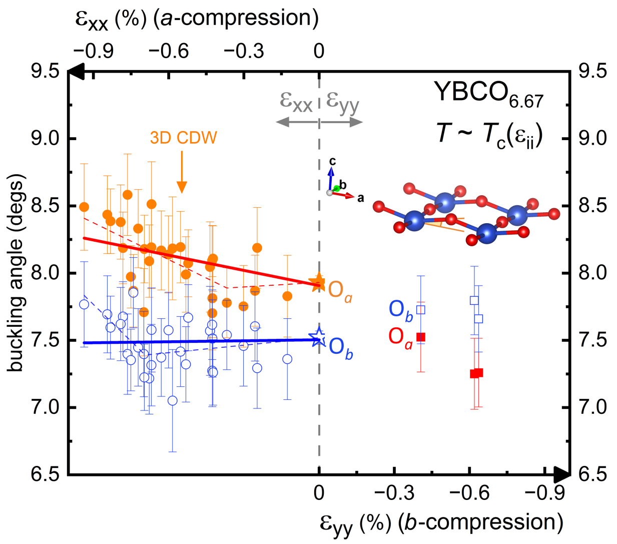

Supplementary Figure 7: (a) Planar oxygen bond buckling angles for a and b axis compression. a-compression amplifies the buckling of the bond along the a axis (filled circles). The thick line is a linear fit. The dashed line is a piece-wise linear fit that has a kink at finite strain. For O_a the kink is consistent within experimental uncertainty to the onset of 3D ordering (arrow). The bond along the b axis (empty circles) is less sensitive to compression when the 3D CDW sets in. Stars refer to single crystal x-ray diffraction at 80 K (see text). Error bars correspond to standard deviations of Rietveld refinements using Jana2020. The inset shows a CuO_2 plane with the buckling angle along the a axis marked by the orange angle.

Supplementary Note 3. Structural information $YBCO_{6.67}$

Refinements of the crystal structure with Jana2020 work best in the geometry for which the transmission axis lies in parallel to an in-plane axis of the unrotated sample (see Supplementary Table 1). All atomic coordinates in the plane are constrained by symmetry, and in this geometry high L-values are accessible which determine the z-coordinates. As noted in the previous section, refinements of the average crystal structure suffer from anomalously high secondary extinction at low strains, but the extinction drops off rapidly for increasing compression. We cross-checked the refinement results with x-ray diffraction at zero strain of a $YBCO_{6.67}$ sample with lab-based XRD on a rotating anode RIGAKU Synergy-DW Mo/Ag system. Using Mo K_α radiation and a resolution of 0.56 Å we measured a T-dependent series. For all temperature steps around 20000 Bragg peaks were used, significantly reducing the experimental uncertainty. Given the very small temperature dependence of the refined parameters between 150 K and the lowest accessible temperature of 80 K, we present the corresponding crystallographic data

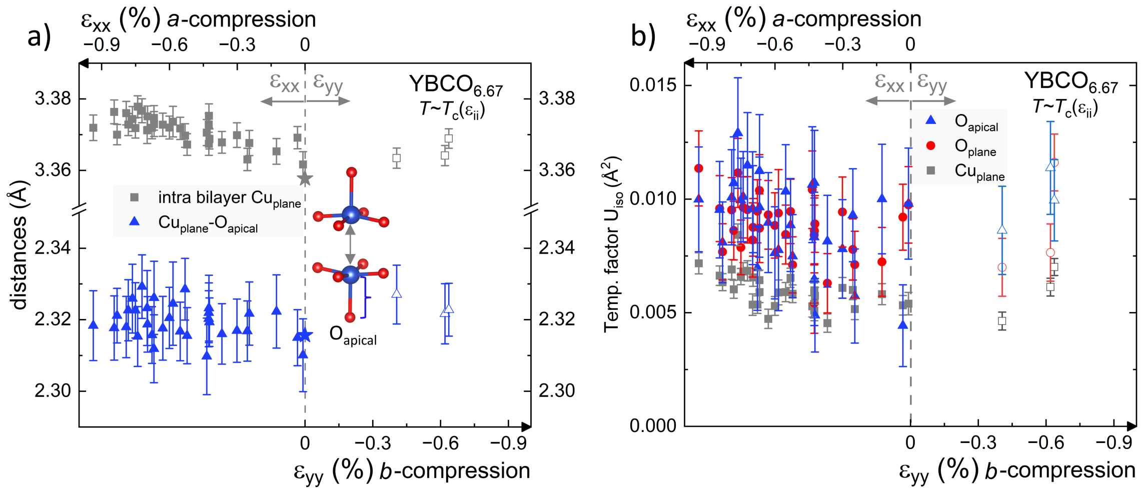

Supplementary Figure 8: (a) Distances of the intra-bilayer distance of planar Cu atoms and the planar Cu to apical O situated above for a and b axis compression. While planar Cu is weakly affected, the apical O does not move as 3D CDW ordering sets in around ε_{xx}=-0.55 %. (b) Isotropic temperature factors of apical and planar O and planar Cu, with no remarkable features larger than the statistical uncertainty. Error bars correspond to standard deviations of Rietveld refinements by Jana2020.

taken at 80 K together with the strain-dependent refinements at $T \sim T_c(\varepsilon_{ii})$ in Supplementary Table 3. In Supplementary Fig. 7 and Supplementary Fig. 8 we display results of structural refinements where we find the largest strain induced changes in the planar oxygen atoms. In Supplementary Fig. 7 1° of buckling corresponds to a vertical displacement of about 3 pm, so the scales in Supplementary Fig. 7 and Fig. 8a are comparable. All refinements (except the cross-check at zero strain) were carried out with isotropic temperature factors and both planar oxygens, O(2) (O_a) and O(3) (O_b), were assumed to possess equal temperature factors, as shown in Supplementary Fig. 8b.

Finally, an interesting structural parameter to be observed under uniaxial strain is the position of the apical oxygen above the Cu bilayer (see Supplementary Fig. 8a). It strongly impacts the intralayer hopping and has been identified as an essential control parameter of the superconducting T_c in cuprates[16]. For instance, the motion of apical oxygen along the c axis, together with a suppression of buckling under intense THz laser pumping have been identified as structural signatures of the high temperature transient superconducting phase of the cuprates[17,18]. Under uniaxial compression, we do not note any change of the apical oxygen position with respect to the CuO_2 plane, nor of the distance between the CuO_2 layers within

	$YBa_2Cu_3O_{6.67}$	unstrained (80 K)	strained (60 K)
	a (Å)	3.8182(1)	3.7883(2)
	b (Å)	3.8764(1)	3.8914(28)
	c (Å)	11.6706(2)	11.6983(4)
Y	U_{eqiv}, U_{iso} (Å^2)	0.0032(1)	0.0067(4)
Ba	z	0.18701(1)	0.18684(7)
	U_{eqiv}, U_{iso} (Å^2)	0.0042(1)	0.0083(4)
Cu(1)	U_{eqiv}, U_{iso} (Å^2)	0.0042(1)	0.0079(5)
Cu(2)	z	0.35615(3)	0.35569(14)
	U_{eqiv}, U_{iso} (Å^2)	0.0028(1)	0.0066(4)
O(1)	U_{eqiv}, U_{iso} (Å^2)	0.0050(9)	0.0222(58)
O(2)	z	0.37893(17)	0.37970(82)
	U_{eqiv}, U_{iso} (Å^2)	0.0049(3)	0.0096(15)
O(3)	z	0.37807(16)	0.37816(84)
	U_{eqiv}, U_{iso} (Å^2)	0.0041(3)	0.0096(15)
O(4)	z	0.15773(15)	0.15757(75)
	U_{eqiv}, U_{iso} (Å^2)	0.0072(4)	0.0095(21)
	GOF	1.24	3.19
	wR_2 (%)	3.15	9.93
	R_1 (%)	0.98	4.08
	extinction	0.025(1)	0.150(30)

Supplementary Table 3: Refinement results for $YBCO_{6.67}$ at zero strain and 80 K, compared to those at strain $\varepsilon_{xx} = -0.845\%$ and 60 K. Due to fewer reflections, the strained samples were refined with isotropic temperature factors (U_{iso}), whereas the unstrained reference measurement was refined with anisotropic temperature factors for which the equivalent isotropic temperature factor (U_{eqiv}) is calculated. Error bars are standard deviations of the Rietveld refinement using Jana2020.

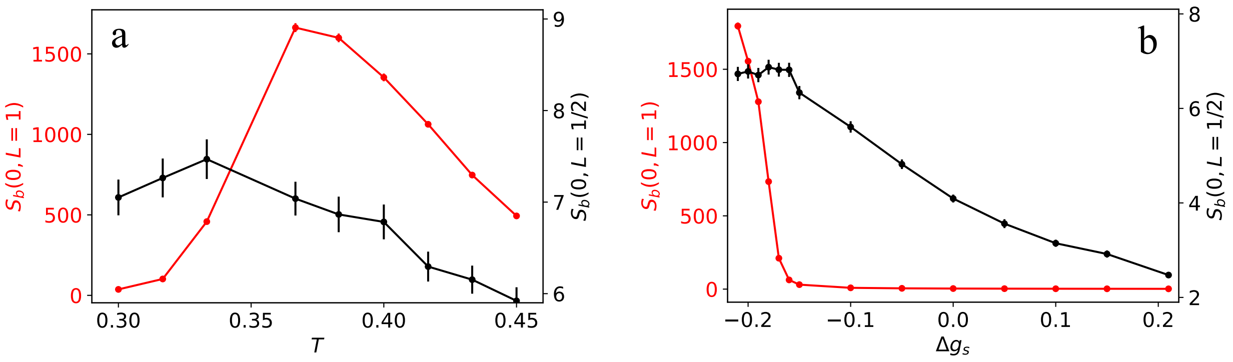

Supplementary Figure 9: The temperature and strain dependence of the b-CDW peaks in a system with the same model parameters as in the main text but with a reduced disorder potential $V = 0.8$. (a) The temperature dependence of the CDW peaks at $L = 1$ and $L = 1/2$ in a system with $\Delta g_s = -0.2$. (b) The Δg_s dependence of the same quantities at $T = 0.383$.

the bilayers. This indicates that a potential structural contribution to the reduction of T_c here is mainly controlled by the changes in the CuO_2 planes.

Supplementary Note 4. Additional theoretical results

In the simulations we have used the set of model parameters that we have previously used in our study of 2D and 3D CDWs in $YBa_2Cu_3O_y$ [9]. Our experience indicates that moderate variations in the values of the parameters do not lead to qualitative changes in the behavior of the model. We have checked that the discs model for the disorder, which we have used in the current study, leads to the same dependence of the CDW signals on the temperature and magnetic field, as found using the Gaussian noise model in Refs. 9,19. In particular, we have found that by reducing the disorder strength one can recover a peak in the temperature dependence of the 2D CDW, as demonstrated in Supplementary Fig. 9. We note that the same figure shows that this change does not qualitatively alter neither the temperature dependence of the 3D CDW nor the strain dependence of the CDWs.

To further characterize the strain dependence of the CDWs we have calculated their in-plane correlation length at a temperature $T = 0.383$, which approximately corresponds to T_c of the model with $\Delta g_s = -0.2$. We observe clear peaks as a function of the in-plane momentum for both $L = 1/2$ and $L = 1$, which we fit to a Lorentzian in order to extract the correlation length of the 2D and 3D CDW, respectively. Supplementary Fig. 10 shows that the while the

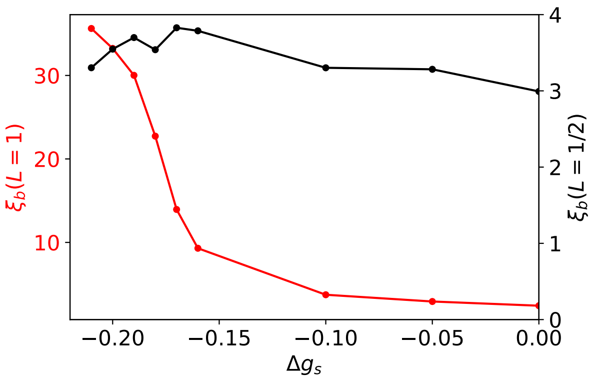

Supplementary Figure 10: The strain dependence of the in-plane correlation length of the b-CDW peaks in a system with the same model parameters as in the main text ($V = 1$) at a temperature $T = 0.383$.

correlation length of the 2D CDW grows very slowly with strain, the correlation length of the 3D CDW rises rapidly beyond its onset, eventually reaching the system size. These findings conform with the experimentally observed strain dependence of the correlation lengths[20].

Supplementary references

---

* These authors contributed equally to this work

† Current address: Institute for Materials Research, Tohoku University, Sendai 980-8577, Japan

‡ Electronic address: matthieu.letacon@kit.edu

1 C. Lin, W. Zhou, W. Liang, E. Schönherr, and H. Bender, Physica C: Superconductivity **195**, 291 (1992), URL https://doi.org/10.1016/0921-4534(92)90353-E.

2 C. Kunz, Journal of Physics: Condensed Matter **13**, 7499 (2001), URL https://doi.org/10.1088/0953-8984/13/34/303.

3 J. Chang, E. Blackburn, A. Holmes, N. B. Christensen, J. Larsen, J. Mesot, R. Liang, D. Bonn, W. Hardy, A. Watenphul, et al., Nat. Phys. **8**, 871 (2012), URL https://doi.org/10.1038/nphys2456.

4 J. Chang, E. Blackburn, O. Ivashko, A. Holmes, N. B. Christensen, M. Hücker, R. Liang, D. Bonn, W. Hardy, U. Rütt, et al., Nat. Commun. **7**, 11494 (2016), URL https://doi.org/10.1038/ncomms11494.

[5] J. Choi, O. Ivashko, E. Blackburn, R. Liang, D. Bonn, W. Hardy, A. Holmes, N. Christensen, M. Huecker, S. Gerber, et al., Nat. Commun. **11**, 1 (2020), URL https://doi.org/10.1038/s41467-020-14536-1.

[6] S. Gerber, H. Jang, H. Nojiri, S. Matsuzawa, H. Yasumura, D. Bonn, R. Liang, W. Hardy, Z. Islam, A. Mehta, et al., Science **350**, 949 (2015), URL https://doi.org/10.1126/science.aac6257.

[7] T. Wu, H. Mayaffre, S. Krämer, M. Horvatić, C. Berthier, P. L. Kuhns, A. P. Reyes, R. Liang, W. Hardy, D. Bonn, et al., Nat. Commun. **4**, 2113 (2013), URL https://doi.org/10.1038/ncomms3113.

[8] S. Nakata, P. Yang, M. E. Barber, K. Ishida, H.-H. Kim, T. Loew, M. L. Tacon, A. P. Mackenzie, M. Minola, C. W. Hicks, et al., npj Quantum Mater. **7**, 118 (2022), URL https://doi.org/10.1038/s41535-022-00532-9.

[9] Y. Caplan and D. Orgad, Phys. Rev. Lett. **119**, 107002 (2017), URL https://link.aps.org/doi/10.1103/PhysRevLett.119.107002.

[10] E. M. Forgan, E. Blackburn, A. Holmes, A. Briffa, J. Chang, L. Bouchenoire, S. Brown, R. Liang, D. Bonn, W. Hardy, et al., Nat. Commun. **6**, 10064 (2015), URL https://doi.org/10.1038/ncomms10064.

[11] G. Grissonnanche, O. Cyr-Choinière, F. Laliberté, S. René de Cotret, A. Juneau-Fecteau, S. Dufour-Beauséjour, M.-E. Delage, D. LeBoeuf, J. Chang, B. J. Ramshaw, et al., Nat. Commun. **5**, 3280 (2014), URL https://doi.org/10.1038/ncomms4280.

[12] R. Zhou, M. Hirata, T. Wu, I. Vinograd, H. Mayaffre, S. Krämer, A. P. Reyes, P. L. Kuhns, R. Liang, W. Hardy, et al., Proc. Nat. Acad. Sci. USA **114**, 13148 (2017), URL https://doi.org/10.1073/pnas.1711445114.

[13] H.-H. Kim, E. Lefrançois, K. Kummer, R. Fumagalli, N. B. Brookes, D. Betto, S. Nakata, M. Tortora, J. Porras, T. Loew, et al., Phys. Rev. Lett. **126**, 037002 (2021), URL https://doi.org/10.1103/PhysRevLett.126.037002.

[14] O. Kraut, C. Meingast, G. Bräuchle, H. Claus, A. Erb, G. Müller-Vogt, and H. Wühl, Physica C: Superconductivity **205**, 139 (1993), URL https://doi.org/10.1016/0921-4534(93)90180-X.

[15] D. LeBoeuf, N. Doiron-Leyraud, B. Vignolle, M. Sutherland, B. J. Ramshaw, J. Levallois, R. Daou, F. Laliberté, O. Cyr-Choinière, J. Chang, et al., Phys. Rev. B **83**, 054506 (2011), URL https:

//doi.org/10.1103/PhysRevB.83.054506.

16 E. Pavarini, I. Dasgupta, T. Saha-Dasgupta, O. Jepsen, and O. K. Andersen, Phys. Rev. Lett. **87**, 047003 (2001), URL https://doi.org/10.1103/PhysRevLett.87.047003.

17 S. Kaiser, Physica Scripta **92**, 103001 (2017), URL https://doi.org/10.1088/1402-4896/aa8201.

18 R. Mankowsky, A. Subedi, M. Först, S. O. Mariager, M. Chollet, H. T. Lemke, J. S. Robinson, J. M. Glownia, M. P. Minitti, A. Frano, et al., Nature **516**, 71 (2014), URL https://doi.org/10.1038/nature13875.

19 Y. Caplan, W. Wachtel, and D. Orgad, Phys. Rev. B **92**, 224504 (2015), URL https://doi.org/10.1103/PhysRevB.92.224504.

20 H.-H. Kim, S. M. Souliou, M. E. Barber, E. Lefrançois, M. Minola, M. Tortora, R. Heid, N. Nandi, R. A. Borzi, G. Garbarino, et al., Science **362**, 1040 (2018), URL https://doi.org/10.1126/science.aat4708.
