# Supplementary material for: Using strain to uncover the interplay between two- and three-dimensional charge density waves in high-temperature superconducting YBa2Cu3Oy
Source: Nat Commun. 2024 Apr 16;15:3277. doi: 10.1038/s41467-024-47540-w (PMC11021565; doi:10.1038/s41467-024-47540-w)
Supplement: Supplementary file 3 — Source Data [file 41467_2024_47540_MOESM3_ESM.zip › READ_ME.pdf]

Supplementary information for software NLSM_monte_carlo

Software requirements

- The software was written in Python and has been tested on Windows 10.
- Before using the NLSM_monte_carlo software, users should have Python version 3.11.7 or higher installed, together with the following packages: numpy, numba, pandas.
- Jupyter Notebook is required for the demo.

Demo

- Save NLSM_monte_carlo and run_NLSM in a directory of your choice. Open run_NLSM using Jupyter Notebook. Set the variable begpath in the first cell to the path of the directory in which you have saved NLSM_monte_carlo and run_NLSM.
- Create a directory to hold the results of the simulation. Set the variable dat_path in cell 3 of the notebook to the path of this directory. Run cells 1-3. For the parameters included in run_NLSM the run time is approximately 9 minutes.
- A folder containing the results is created in the folder that you have created in the previous item. To view the results set the variable res in cell 4 to the path of the directory created by the simulation, and run the cell.

Calling NLSM_monte_carlo

Input parameters involved in setting up the Monte Carlo simulation:

- dat_path - (str) Holds the path of the directory, where the results are to be saved.
- L - (int) The in-plane size of the system. Each plane contains $L \times L$ sites.
- Lz - (int) The number of planes. This should be an even number since the model consists of bilayers.
- N_sweep - (int) Total number of Monte Carlo sweeps.
- N_skip - (int) Number of sweeps for thermalization.
- corr_t - (int) Number of sweeps between consecutive measurements.
- seed - (int) Seed for random number generation.

Input parameters defining the NLSM model (Eqs. 2 and 3 of the main text). The code assumes $\rho_s = 1$ and $\lambda = 1$:

- temperature - (float) Temperature (T).
- mag_field - (float) The magnetic field (not used for the purpose of the present study).
- g - (float) The effective CDW mass (g).
- dg - (float) The CDW mass anisotropy due to the chains (Δg).
- dg_strain - (float) The CDW mass anisotropy due to the strain (Δg_s).
- U_int - (float) The intra-bilayer CDW interaction coupling ($\tilde{U}$).
- U_int_bi - (float) The inter-bilayer CDW interaction coupling (U).

- J_int - (float) The intra-bilayer Josephson coupling ($\tilde{J}$).
- J_int_bi - (float) The inter-bilayer Josephson coupling (J).
- w - (float) Set to zero. Not used in the present model.
- V - (float) The amplitude of the disorder within a disc (V).
- V_local - (float) The code allows to include also disorder on the planes in addition to the disorder on the chain layers (see. Ref. 39 in the main text). The in-plane disorder is modeled as random Gaussian fields whose standard deviation is given by V_local.
- gamma - (float) The ratio between the coupling to the disorder on the inner and outer planes (γ).
- d_length - (int) The radius of the disorder discs (r_d).
- N_defect - (int) The number of disorder discs per bilayer.
- onsite - (bool) False if the disorder discs are to be centered on centers of plaquettes. True if the discs are to be centered on sites.

Output of NLSM_monte_carlo

The output is a dictionary that contains the input parameter, and in addition:

- disorder_config - A 4-dimensional array of size $L \times L \times L_z \times 4$ containing the total disorder potential to which the CDW fields Φ^α, $\alpha = 1, 2, 3, 4$ couple on the site (x, y, z). The fields Φ^1, Φ^2 correspond to the real and imaginary parts of the complex field Φ^b of the main text, while Φ^3, Φ^4 correspond to the real and imaginary parts of Φ^a.
- G_ab - A 6-dimensional array of size $6 \times L \times L \times L_z/2 \times 2 \times 2$ containing the correlations

$$G^\alpha_{\mu\mu'}(\mathbf{q}, q_z) = \sum_{\mathbf{rr'}} \sum_{jj'} e^{-i\left[\mathbf{q}\cdot(\mathbf{r}-\mathbf{r}') + q_z(j-j')\right]} \langle \phi^\alpha_{j\mu}(\mathbf{r}) \phi^\alpha_{j'\mu'}(\mathbf{r}') \rangle,$$

  where $\phi^1 = \mathrm{Re}(\psi)$, $\phi^2 = \mathrm{Im}(\psi)$, and $\phi^\alpha = \Phi^{\alpha-2}$ for $\alpha = 3, 4, 5, 6$. The array indices stand for $(\alpha, q_x, q_y, q_z, \mu, \mu')$. The momenta take the values $q_{x,y} = \frac{2\pi}{L} n_{x,y}$, where $n_{x,y} = 0, \cdots, L-1$, and $q_z = \frac{4\pi}{L_z} n_z$ with $n_z = 0, \cdots, \frac{L_z}{2} - 1$.
- n_a - A 4-dimensional array of size $L \times L \times L_z \times 6$ containing $\langle [\phi^\alpha(x, y, z)]^2 \rangle$.
- helicity - A 1-dimensional array of size (N_sweep − N_skip)/corr_t containing the measurements of the superconducting stiffness calculated in the course of the simulations.
- order - A 4-dimensional array of size $L \times L \times L_z \times 6$ containing the final Monte Carlo measurement of the fields $\phi^\alpha(x, y, z)$.
